# Could SARS-CoV-2 Have Bacteriophage Behavior or Induce the Activity of Other Bacteriophages?

**DOI:** 10.3390/vaccines10050708

**Published:** 2022-04-29

**Authors:** Carlo Brogna, Barbara Brogna, Domenico Rocco Bisaccia, Francesco Lauritano, Giuliano Marino, Luigi Montano, Simone Cristoni, Marina Prisco, Marina Piscopo

**Affiliations:** 1 Department of Research, Craniomed Group Facility Srl., 20091 Bresso, Italy; roccobisa@gmail.com (D.R.B.); francescodrlauritano@gmail.com (F.L.); 2 Department of Radiology, Moscati Hospital, Contrada Amoretta, 83100 Avellino, Italy; brognabarbara1@gmail.com; 3 Marsan Consulting Srl., Public Health Company, Via dei Fiorentini, 80133 Naples, Italy; marino@marsanconsulting.it; 4 Andrology Unit and Service of Life Style Medicine in Uro-Andrology, Local Health Authority (ASL), 84124 Salerno, Italy; luigimontano@gmail.com; 5 ISB—Ion Source & Biotechnologies Srl., 20091 Bresso, Italy; simone.cristoni@gmail.com; 6 Department of Biology, University of Naples Federico II, 80126 Naples, Italy; marina.prisco@unina.it

**Keywords:** SARS-CoV-2, human microbiota, Sabin vaccine, electron microscope, mucosal immunity, bacteriophage

## Abstract

SARS-CoV-2 has become one of the most studied viruses of the last century. It was assumed that the only possible host for these types of viruses was mammalian eukaryotic cells. Our recent studies show that microorganisms in the human gastrointestinal tract affect the severity of COVID-19 and for the first time provide indications that the virus might replicate in gut bacteria. In order to further support these findings, in the present work, cultures of bacteria from the human microbiome and SARS-CoV-2 were analyzed by electron and fluorescence microscopy. The images presented in this article, in association with the nitrogen (^15^N) isotope-labeled culture medium experiment, suggest that SARS-CoV-2 could also infect bacteria in the gut microbiota, indicating that SARS-CoV-2 could act as a bacteriophage. Our results add new knowledge to the understanding of the mechanisms of SARS-CoV-2 infection and fill gaps in the study of the interactions between SARS-CoV-2 and non-mammalian cells. These findings could be useful in suggesting specific new pharmacological solutions to support the vaccination campaign.

## 1. Introduction

Coronavirus disease 2019 (COVID-19) is having a devastating effect on the world’s population, as it has caused more than 5.4 million deaths worldwide. For this reason, this disease is recognized as the most significant global health crisis since the 1918 influenza pandemic. Since the World Health Organization (WHO) declared a global pandemic on 11 March 2020, many countries continue to experience multiple waves of outbreaks of this viral disease.

Currently, a new variant has already arrived from South Africa: “Omicron.” The data officialized by the WHO as of 7 April 2021, are as follows: 493,392,853 confirmed cases; 6,165,833 deaths; and as of 4 April 2022, over 11 billion COVID-19 vaccine doses had been administered worldwide [1]. The vaccines administered are almost half of the “classic” vaccines based on inactivated viruses (CoronaVac produced by Sinovac Biotech Ltd., Beijing, China), and half of the latest generation of vaccines based on adenoviral vectors (AZD1222 produced by AstraZeneca, Oxford, UK, Ad26.COV2.S by Janssen Pharmaceuticals, Beerse, Belgium) or mRNAs (mRNA-1273 by Moderna, BNT162b2 by Pfizer—BioNTech) [1]. Many studies since the beginning of the pandemic have focused on the interaction between the ACE2 receptor and the virus spike glycoprotein [2], how the enzyme furin works, and how it plays a critical role between the eukaryotic host cell and the viral particle [3]. These observations are derived from past knowledge of SARS [4] and MERS [4]. However, has it been tested whether these RNA viruses, including coronaviruses, can also replicate in cells other than the mammalian eukaryotic cell? In addition, has it been investigated whether they also replicate in yeast or bacteria? [5]. Our previous work showed that people with COVID-19 had toxin-like peptides, which were almost identical to the toxic components of animal venoms, such as conotox, phospholipases A2, phosphodiesterases, zinc metal proteinases, and bradykinins, in both blood, feces, and urine [6]. In a later study, we observed spontaneous replication of SARS-CoV-2 in bacterial cultures from patient feces for up to 30 days and beyond [7,8]. For that matter, there have also been other researchers who have studied the relationship between the gut microbiota and COVID-19. Wang et al., 2021, for example, found significant alterations in the gut and respiratory microbiome in COVID-19 patients, finding that there was an increase in pathogenic bacteria and a decrease in beneficial ones [9]. In addition, to further understand microbial function in the lower respiratory tract in COVID-19 infection, a research group of San Jose, CA, USA, conducted a functional analysis of previously published metatranscriptome sequencing data of bronchoalveolar lavage fluid from COVID-19 cases, patients with community-acquired pneumonia, and healthy controls [10]. By investigating related metabolic pathways, distinguishable functional signatures in COVID-19 respiratory tract microbiomes were revealed, including decreased potential for lipid metabolism and glycan biosynthesis and metabolism pathways, and enhanced potential for carbohydrate metabolism pathways. The findings also suggested new connections to consider, possibly specific to the lower respiratory tract microbiome, requiring further research on microbial function and host-microbiome interactions during SARS-CoV-2 infection [10]. Another very interesting finding emerged from a bioinformatics analysis that revealed that some members of commensal upper respiratory tract (URT) bacteria express proteins that bind the SARS-CoV-2 spike glycoprotein, for example the ACE2-like protein. Based on this analysis and available data showing a decline in the population of these bacteria in the elderly, it has also been proposed that some commensal bacteria of the upper respiratory tract prevent SARS-CoV-2 infectivity and that a decrease in these bacteria contributes to infection severity [11]. The same authors, in another study observed that N501Y mutations in the receptor-binding domain (RBD) of the SARS-CoV-2 spike glycoprotein may allow increased binding to the ACE2 receptor when natural products (NPs) of upper respiratory tract bacteria are present [12]. In addition, there is also another thing to consider. Bacteriophages are viruses that infect bacterial cells. These viruses have long been considered neutral to animals and humans because specific receptors for bacteriophages on eukaryotic cells are lacking. However, very recent studies have provided clear evidence that bacteriophages can interact with eukaryotic cells, causing effects on the functions of the immune system, respiratory system, central nervous system, gastrointestinal system, urinary tract, and reproductive system [13].

Given these assumptions, in order to add new knowledge to the understanding of the mechanisms of SARS-CoV-2 infection and to fill the gaps in the study of the interactions between SARS-CoV-2 and non-mammalian cells, in the present work, bacterial and SARS-CoV-2 culture preparations, obtained from the experiment published in our first work [7], were analyzed by electron and fluorescence microscopy. The aim of our study was to elucidate the interaction between SARS-CoV-2 and human gut microbiota. The data will help to suggest specific therapeutic approaches to support the current vaccines and improve the vaccination campaign.

## 2. Materials and Methods

In vitro bacterial cultures were obtained from fecal (stool) samples from subjects both SARS-CoV-2 positive and negative (for control cases) for viral infection. Informed consents from patients were obtained according to local legislation. Replication of virus in human bacteria by statistical analysis, Luminex molecular tests, and further controls by the mass spectrometry technique (SACI technique) are reported in Petrillo et al., 2021 [7]. Immunogold was performed with the bacteria culture pellet at day 30 after centrifugation of the aliquots. Supernatants were used from the same samples to acquire immunofluorescent images using a fluorescence microscope (100× magnification, Zeiss Axioplan 2, Axiocam 305 color Carl Zeiss Microscopy GmbH, Carl-Zeiss-Promenade 10, 07745 Jena, Germany). Each step was repeated three times.

### 2.1. Electron Microscopy

For electron microscopy analyses, aliquots of bacterial pellets (0.5 mL) obtained after 30 days of bacterial cultures were fixed by resuspending them in 1% glutaraldehyde. They were then centrifuged at 10,000 rpm for ten minutes, harvested as pellets, fixed in 2% OsO_4,_ and processed according to the standard procedures, by dehydration in ethanol followed by infiltration and embedding in Epon resin and polymerization at 60 °C for 48 h. Then, 60-nm thin sections were cut from the sample using a Reichert-Jung ultramicrotome. Grids were counterstained with UranyLess EM stain and Lead Citrate 3% (Electron Microscopy Science). Images were obtained from thin sections under an electron microscope (Tecnai G2 Spirit BioTwin; FEI, Hillsboro, OR 97124-5793 USA) equipped with a VELETTA CCD digital camera (Soft Imaging Systems GmbH, Johann-Krane-Weg 39, 48149 Münster, Germania).

### 2.2. Immunogold Labelling Tecnique

Another pellet of 0.5 mL of the same bacterial cultures was fixed by resuspending with 0.05% glutaraldehyde and 4% paraformaldehyde in 0.1 M PBS buffer, and then centrifugated to 10,000 rpm for ten minutes. Thereafter, the bacteria pellets were post-fixed in 1% OsO_4,_ and processed according to the standard procedures, by dehydration in ethanol followed by infiltration and embedding in Epon resin and polymerization at 60 °C for 48 h. Ultrathin (60-nm) sections were cut from the sample using a Reichert-Jung ultramicrotome and collected onto Formvar/Carbon coated nickel grids. After etching with 3% sodium meta periodate and blocking with 3% BSA in PBS buffer, the samples were incubated in a primary rabbit monoclonal [EPR24334-118] to SARS-CoV-2 nucleocapsid protein antibody (Abcam ab 271180) 1:1000 in 1% BSA in PBS buffer overnight at 4 °C and then in anti-rabbit 10 nm gold-conjugated secondary antibody (Aurion) 1:20 in PBS buffer for 2 h at room temperature.

A negative control was obtained by omitting the primary antibody and using only the secondary gold-conjugate antibody. The control of immunogenicity of antibodies primary versus nucleocapsid protein of SARS-CoV-2 was assumed from Shang C. et al. [14].

Grids were counterstained with UranyLess EM stain and Lead Citrate 3% (Electron Microscopy Science) and images were acquired under a transmission electron microscope (FEI Tecnai G2 S-TWIN, Hillsboro, OR 97124-5793, USA, equipped with a bottom-mounted FEI Eagle4k digital camera.

### 2.3. Validation of Antibody against Nucleocaspid-Protein of SARS-CoV-2 with Fluorescent Microscopy

cDNA encoding green fluorescent protein (GFP)-tagged N-protein of SARS-CoV-2 was generously provided by Dr. M.A. De Matteis (TIGEM, Pozzuoli, Italy) and transfected into HeLa cells using TransIT-LT1 reagent (Mirus Bio LLC, Madison, 53719 WI, USA) according to the manufacturer’s instructions. Transfected cells were fixed in 4% paraformaldehyde, washed with PBS, and incubated in blocking solution (50 mM NH_4_Cl, 0.1% saponin, 1%BSA in PBS). Then, the cells were labelled using a primary antibody against N protein (Abcam, #ab273167) and secondary anti-rabbit IgG conjugated with Alexa Fluor 568 (Thermo Fisher; # A-11011). After labelling, the samples were examined under a LSM700 confocal microscope (Zeiss). Efficiency of the antibody against the N-protein was confirmed based on the presence of Alexa Fluor 568 signal in the cells expressing GFP-tagged N-protein.

### 2.4. Immunofluorescence

Aliquots of the supernatants of the samples were obtained and the slices were fixed with 4% PFA for 5 min. After permeabilization with Triton 0.3% in PBS for 10 min, the slices were rinsed in PBS and blocked with BSA 1% and saponin 0.05% for 30 min. The slices were incubated with the primary antibodies for 2 h at room temperature and then with secondary antibodies for 1 h at room temperature. Immunofluorescence was performed according to the manufacturer’s protocol using a primary antibody against the virus nucleocapsid protein (“Sars Nucleocapsid Protein Antibody [Rabbit Polyclonal]—500 μg 200-401-A50 Rockland”), the “Goat anti-RabbitIgG (H + L) Cross-Adsorbed Secondary Antibody, Cyanine3 #A10520” as a secondary antibody, and a primary antibody against Gram-positive bacteria (“Gram-Positive Bacteria Ab (BDI380), GTX42630 Gene Tex”) and “Goat anti-Mouse IgG (H + L), Super-clonal™ Recombinant Secondary Antibody, AlexaFluor 488”( Life Technologies, Carlsbad, CA, USA) as a secondary antibody”. The negative control of the bacterial cultures was performed without primary antibodies versus Gram-positive bacteria and without primary antibodies versus nucleocapsid SARS-CoV-2 proteins. The control of immunogenicity of primary antibodies versus Gram-positive bacteria was performed on the culture negative at Luminex molecular test to SARS-CoV-2. It was also assumed that the control of immunogenicity of antibodies versus Gram-positive bacteria reflected that from Kohda et al. and Kameli et al. [15,16]. The control of immunogenicity of primary antibodies versus the nucleocapsid protein of SARS-CoV-2 was assumed from Zhao et al. [17].

### 2.5. Proteomic Profiling Analysis

One bacterial liquid culture was used to add, at the end of 30 days, 0.20 g of a nitrogen isotope (^15^N) labeled medium (Sigma Aldrich, St. Louis, MO, USA). After another 7 days, a SARS-CoV-2 proteomic analysis was performed on aliquots (0.2 mL) of the same samples by SACI technology [18]. Modified peptides of a nucleocapsid SARS-CoV-2 protein were detected by extracting the species ^13^C n^15^N (where *n* > 1). Ion proton rearrangement reaction occurring in Collisional Induced Dissociation (CID) conditions were considered in the database search data elaboration [19]. The ion was isolated using 5 *m*/*z* units and the fragmentation energy was 35% of its maximum value (5V peak to peak).

### 2.6. NGS Sequencing Data

DNA from fecal samples, at day 0 (B0) was extracted with E.Z.N.A.^®^ Stool DNA Kit (Omega Bio-Tech, Norcross, GA, USA). Two types of kit for the nucleic acid extractions from samples after 30 days (B1) of culture were used: MasterPure Complete DNA and RNA purification kit (Lucigen, WI, USA) and PureLink Viral RNA/DNA kit (Thermo Fisher Scientific, Waltham, MA, USA). All extractions were performed following the manufacturers’ recommendations. The Ovation^®^ Ultralow V2 DNA-Seq Library Preparation kit (NUGEN, San Carlos, CA, USA) was used for library preparation, following the manufacturer’s instructions. Both input and final libraries were quantified with the Qubit 2.0 fluorometer (Invitrogen, Carlsbad, CA, USA), and the quality was tested by the Agilent 2100 Bioanalyzer High Sensitivity DNA assay (Agilent Technologies, Santa Clara, CA, USA). Libraries were then prepared for sequencing and sequenced on NovaSeq 6000 in 150-bp paired-end mode. Bioinformatic reconstruction was calculated on the fasta file of bacterial sequences on UniProt. The results are presented in Table S1, in Supplementary Material.

## 3. Results

### 3.1. Replication and Viral Load Increase of SARS-CoV-2 in Bacterial Cultures

**Figure 1:**
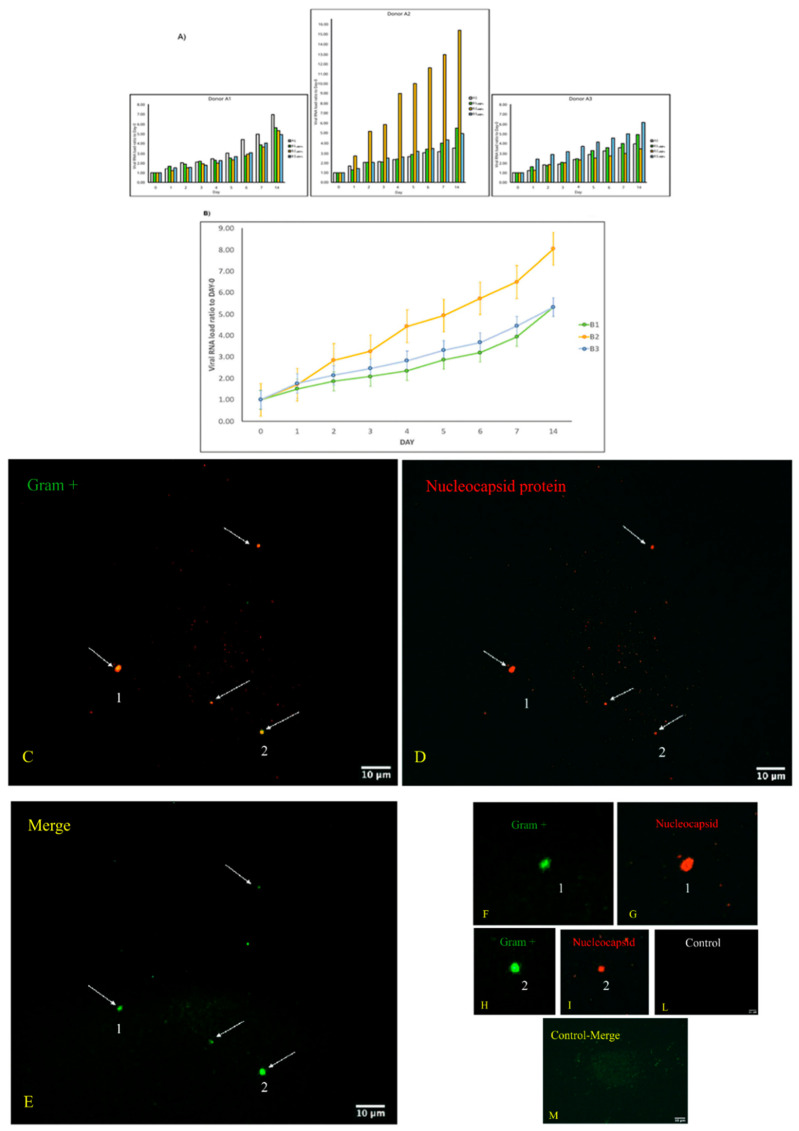
Panel (**A**,**B**): viral increase of SARS-CoV-2 in bacterial cultures. These images are reused and correspond to Figure 3 of Petrillo et al., 2021 obtained with the gentle permission of Dr. Petrillo et al., 2021 [7]. Results of experiments combining samples from three “infected donor” sources (A1–A3) and from three “healthy recipient” sources (B1–B3). (**A**) The graphs report results of nine combinations. To normalize the measurements, all values at day 0 were used as the denominator (at day 0 all values = 1), i.e., for each sample, at day X, the ratio between Luminex Count at DayX/Luminex count at day 0 was calculated. Each bar represents the SARS-CoV-2 RNA load ratio. (**B**) Each line represents the average of the SARS-CoV-2 RNA load ratio of samples B1 (green line), B2 (yellow line), and B3 (azure line) infected each one with three different A donor sources. To normalize the measurements, all values at day 0 were normalized as described in panel (**A**). Panels C-L show the immunofluorescence microscope images (magnification 100×, Zeiss Axioplan 2, Axiocam 305 color, Carl Zeiss Microscopy GmbH, Carl-Zeiss-Promenade 10, 07745 Jena, Germany) on bacterial cultures (performed at 30 days) from the patients positive to SARS-CoV-2 with anti-Gram-positive bacteria (green signal, (**C**)) and anti-SARS-CoV-2 nucleocapsid protein (red signal, (**D**)) antibodies. (**E**): merge of C and D images, particles positive to both antibodies were indicated by white arrows. (**F**–**I**): computerized magnification of particles numbered in (**C**–**E**). Panel (**L**): negative control by omitting primary antibodies. Panel (**M**) is the merge of the negative control of a healthy fecal bacterial culture, in which the anti-bodies versus nucleocapsid protein did not give signal and Gram+ bacteria are present. See text for more details.

The curves of replication and viral load increase of SARS-CoV-2 in bacterial cultures are shown in Figure 1, Panels A and B, with the permission of Dr. Petrillo [7]. In particular, three couples of fecal samples from different “infected donors” (i.e., sources of A) and “healthy recipients” (i.e., sources of B) have been recruited, and subject to the same experimental procedure described in the Materials and Methods section. All combinations of “infected donors” sources (A1, A2 and A3) and “healthy recipients” donors (B1, B2 and B3) were subject to the same experimental procedure. Although with certain differences, the observed trends were similar (Figure 1A), confirming the increase over time of SARS-CoV-2 RNA load in samples of type A and in samples of type B (A+). Lastly, for each “recipient”, SARS-CoV-2 RNA load was measured (Figure 1B). The results report virus replication in bacterial cultures suggesting that this virus behaves as a bacteriophage.

### 3.2. Microscopic Analysis of the Interaction between SARS-CoV-2 and the Human Microbiome

To deepen our previous studies [6,7], analyses using immunofluorescence, TEM, and immunogold marking techniques were conducted. These analyses showed infection of bacteria by the SARS-CoV-2 virus after 30 days of bacterial culture, where mammalian eukaryotic cells are excluded, in agreement with current literature that excludes their survival beyond 48 h [20]. Culture conditions have been described previously (see Materials and Methods by Petrillo et al. [7]).

Double immunofluorescence was conducted using an anti-GramPositive Bacteria Marker and an anti-SARS-CoV-2 nucleocapsid protein and this experiment showed co-localization of both signals on bacterial cells (Figure 1). In particular, the green signal corresponds to the Gram-positive bacteria that are shown in the images in panels C, F, H. The red signal indicates the nucleocapsid protein of SARS-CoV-2. In panels D, G, and I, the same bacteria shown in the previous panels are shown. Since these are labeled with this signal, it means that they are infected with the virus. The confirmation is obtained from the overlay/merge fluorescence shown in panel E in which the Gram-positive bacteria (signal with green light- in panels C, F, and H) were attached to viruses like-particles of SARS-CoV-2 (signalled with red light, in panel D, G, and I). In panels F and H, it is possible to observe the computerized magnifications of the bacteria indicated with numbers 1 and 2 that appear green because they are marked with the anti Gram-positive antibody. Similarly, panel G is a computerized magnification of the bacteria attached by SARS-CoV-2 indicated with the numbers 1 and 2. The negative control of the culture obtained without primary antibodies but only with secondary antibodies is shown in panel L. Panel M is the merge of the negative control of a healthy fecal bacterial culture, in which the antibodies versus nucleocapsid protein did not give a signal and Gram-positive bacteria are present.

**Figure 2:**
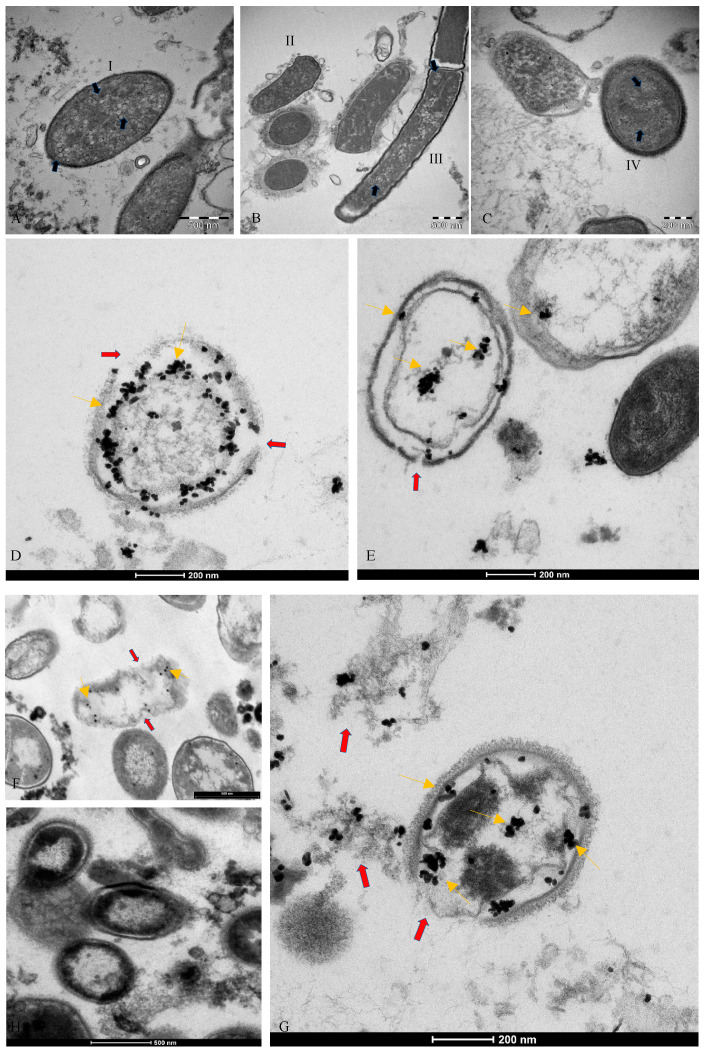
TEM images of bacterial cultures from SARS-CoV-2 positive patients. (**A**–**C**) shows circular structures (blue arrows) inside bacteria cells (I, II, III, IV). (**D**–**G**): immunogold labelling technique on the same samples with anti-nucleocapsid protein of SARS-CoV-2 antibody (gold arrows); the gold particles are inside the bacteria. Red arrows show lysis of the bacteria membrane. Specifically, image (**G**) shows bacteria wall membrane lysate (red arrow) and the antibody binding to SARS-CoV-2 N protein in the cell (gold arrows). (**H**): negative control. See text for more details.

An analysis performed by transmission electron microscopy, showed virus-like particles near and within the bacteria as can be seen in Figure 2A–C. In addition, immunogold labeling performed with an anti-SARS-CoV-2 nucleocapsid protein antibody revealed the gold nanoparticles in the bacteria (Figure 2D–G). Initial areas of cell wall lysis and fully lysed bacteria are also observed (indicated in Figure 2 with red arrows). Panel H represents the negative immuno-gold control. No mammalian eukaryotic cells are present after 30 days of bacterial cultures incubated under the conditions previously described in Petrillo et al. [7]. The antibody specificity is shown in Supplementary Figure S1A–D. The presence of gold nanoparticles in bacteria was also observed by performing immunogold pre-embedding in another facility, using an anti-SARS-CoV-2 nucleocapsid protein antibody. This analysis revealed that only one yeast was found in the culture after 30 days, and no viral particles were present (Supplementary Figure S1 panel E–F). Visualization of gold nanoparticles in bacteria are clear evidence that the virus is in the bacteria.

### 3.3. NGS Sequencing Data

**Figure 3:**
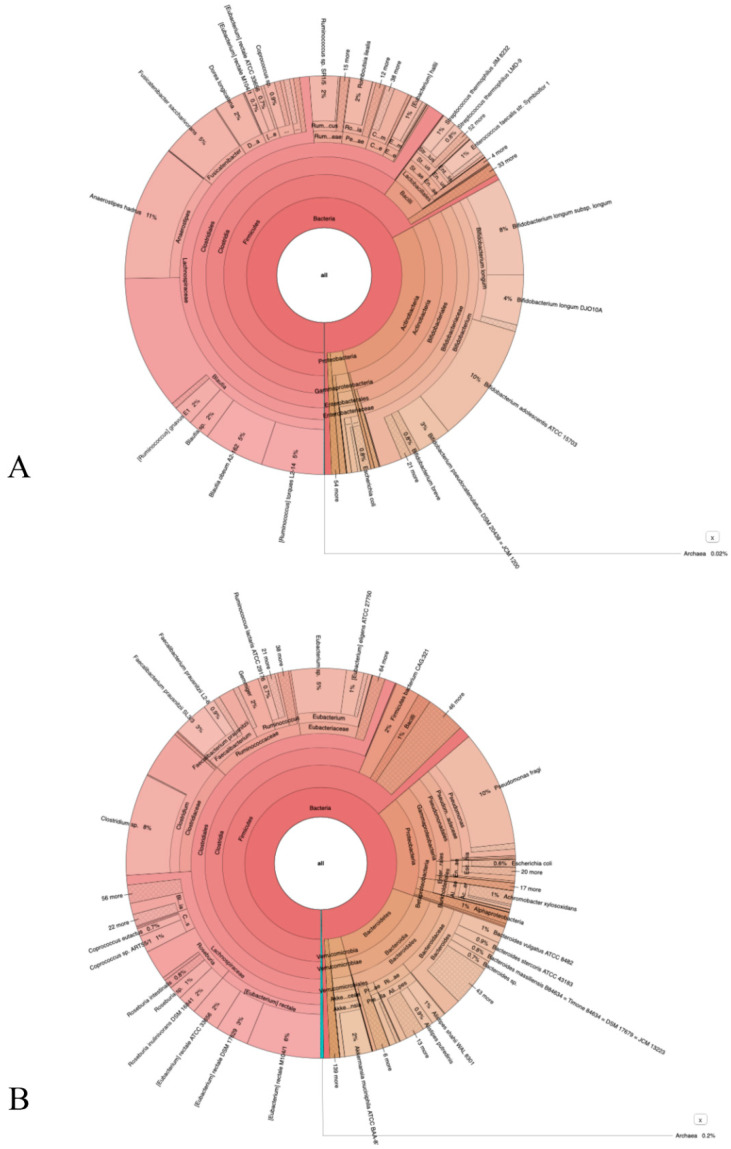
Panel (**A**): represents the phylum of bacteria present in the culture before the addition of SARS-CoV-2, while panel (**B**) represents the change in bacterial population after 30 days of the same cultures infected with SARS-CoV-2. For more detail see supplementary materials.

An interactive metagenomic visualization using the tool Krona [21] is shown in Figure 3A,B. The multilayer pie chart represents the degrees of the bacterial world. The division is from inside to outside the circle. Colors towards red, have a low level of confidence while colors towards green have a high level of confidence. The initial and final samples, after 30 days, of the bacterial cultures are compared. The genera of bacteria such as *Dorea*, *Fusicatenibacter*, *Klebsiella*, and *Streptococcus* decreased while other genera of bacteria such as *Campylobacter*, *Prevotella*, *Staphylococcus*, *Bacteroides*, and *Cytobacter* increased.

### 3.4. Nitrogen Isotope ^15^N Assay in Bacterial Culture Medium. Proteomic Profiling Analysis

**Figure 4:**
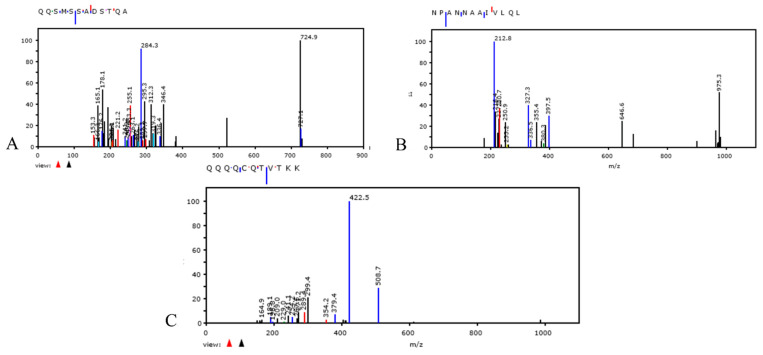
Panels (**A**–**C**): show the MS/MS spectra of the nucleocapsid protein of SARS-CoV-2 containing the nitrogen isotope. Panel (**A**) shows peptide seq: QQSMSSADSTQA; ID:|A0A8B1JYE4|A0A8B1JYE4_SARS2; Mods: Q408 + 1 (^13^C) + 2 (^15^N), Q409 + 1 (^13^C) + 2 (^15^N). Panel (**B**) shows peptide seq: NPANNAAIVLQL; ID: A0A7M1YDW6|A0A7M1YDW6_SARS2; Mods: Q160 + 1 (^13^C) + 2 (^15^N). Panel (**C**) shows peptide seq: QQQQCQTVTKK; ID:|A0A8B1XSI6|A0A8XSI6_SARS2; Mods: Q239 + 1 (^13^C) + 2 (^15^N), Q239+Ammonia-loss, Q240 + 1 (^13^C) + 2 (^15^N), Q241 + 1 (^13^C) + 2 (^15^N), Q242 + 1 (^13^C) + 2 (^15^N), Q244 + 1 (^13^C) + 2 (^15^N).

After 30 days of bacterial growth in liquid medium, nitrogen (^15^N) isotope-labeled medium (Sigma Aldrich, St. Louis, MO, USA) was added. After an additional 7 days, proteomic analysis of SARS-CoV-2 was performed. Modified peptides of the SARS-CoV-2 nucleocapsid protein were detected by extracting the ^13^C n^15^N species as specified in the Materials and Methods section. The spectra obtained by Tandem mass spectrometry (MS/MS) [18] and acquired between 200 and 1800 *m*/*z* (mass/charge), show fragmentation of the modified peptide of nucleocapsid SARS-CoV-2 protein, containing ^13^C and ^15^N isotopes. The sequences are shown in Figure 4A–C. The nitrogen isotope (^15^N) was detected in a nucleocapsid peptide of SARS-CoV-2 replicated in bacterial cultures by monitoring the molecular mass shift. The MS/MS spectra shows that the ^15^N species is located on amino acid residue of peptides. Nitrogen (^15^N) isotope labeling in SARS-CoV-2 proteins demonstrates that bacteria can replicate, transcribe, and translate viral RNA and thus that bacteria produce the proteins of this virus.

## 4. Discussion

In an effort to counter the current global pandemic, characterization of the molecular-level interactions with the host of the novel SARS-CoV-2 coronavirus that causes COVID-19 has become the subject of much scientific attention. However, when the virus enters the body it also interacts with microorganisms that already inhabit the host. Understanding virus-host-microbiome interactions may provide additional insight into the biological processes perturbed by viral invasion. Sifting through old scientific literature can be challenging and valuable. For example, some past studies have observed a co-participation of bacteria in the infection of another RNA virus such as HIV [22]. In addition, recent studies have observed the presence of HIV in intestinal mucosal lymphoid tissues [23].

In a recent case report, it was reported that 500 days after recovering from Ebola virus (fecal-oral RNA virus), an African boy sexually transmitted the virus to a woman [24]. One would wonder: how is this possible? Where was the Ebola virus hiding?

It should be kept in mind that many studies have observed that SARS-CoV-2 is almost always present in the feces of sick and asymptomatic individuals, suggesting a fecal-oral route of transmission [25]. In addition, its closest relatives (coronavirus RATG13) have been found in the feces of bats [5]. Experience with SARS-CoV-2 also suggests this route of transmission [26]. During outbreaks of other coronaviruses, CoV-OC-43 and CoV-NL-63, district-like epidemics of these viruses have been reported, with the same symptoms and epidemic statistics as SARS-CoV-2 [27]. The coronaviruses of pigs and cattle also show a circumscribed transmission pattern [28]. If we look more closely at the history of poliovirus (yet another RNA virus), it was only after many years that it became clear that its transmission was not exclusively airborne, as was mistakenly believed, but also fecal-oral [29]. In fact, observing the epidemiology and spread of polioviruses, it is evident that they have always caused district epidemics but have never generated a pandemic crisis. It is also well known that a problem faced by the scientific community was the procurement of sterile artificial steel lungs for hospitals to contain their spread [30].

In 1956, Albert B. Sabin indicated the pathogenesis of poliomyelitis, which had been debated until then “*Polio is an RNA virus with fecal-oral transmission. Its significant replication occurs in the intestinal tract*” [31,32,33,34].

Interestingly, at that time, a group of researchers observed how bacteria might play a role in the selection of a few different strains of poliovirus. They observed that bacterial endotoxins induced a lower rate or even inhibited viral replication [35]. Similar to our studies [6,7], we highlighted several toxin-like proteins in the plasma, feces, and urine of COVID-19 patients, which were produced by bacteria as a consequence of interaction with SARS-CoV-2. We have shown that these oligopeptides are derived from bacterial cultures [6,7] and we are testing them in mice (data in preparation). We have already observed the replication of SARS-CoV-2 virus in bacteria many times [7] and have conducted some experiments with Nitrogen Isotope ^15^N in the bacteria culture in the presence of this virus to better understand this phenomenon (Figure 4). This finding is reported in another of our works, concerning the spike protein and the nitrogen isotope present in it [36]. Furthermore, we found that after 30 days of bacterial cultures, using a previously published methodology [7], some bacterial genera tend to increase and others tend to decrease. These data suggest that the increase in viral load, as previously shown by Petrillo et al. [7], in bacterial cultures, is associated with a decrease in some bacterial genera after 30 days, perhaps is due to the lytic cycle of these bacteria (Table S1 in Supplementary Material). We found that some bacterial genera, such as *Dorea*, *Fusicatenibacter*, *Klebsiella*, and *Streptococcus* decreased, while other genera, such as *Campylobacter*, *Prevotella*, *Staphylococcus*, *Bacteroides*, and *Cytobacter* increased. Our results are in line with those of Liu et al., 2021 [37] who performed a metabolomic analysis of blood, urine, and nasopharyngeal swabs of a COVID-19 and non-COVID-19 patient group and a metagenomic analysis of pharyngeal samples. Their results indicated that nasopharyngeal commensal bacteria including *Gemella morbillorum*, *Gemella haemolysans*, and *Leptotrichia hofstadii* were significantly depleted in the pharynges of COVID-19 patients, whereas bacteria such as *Prevotella histicola*, were relatively increased. In addition, the mechanism of interaction that we have observed could be very similar to that previously reported by Ebrahimi, 2020 [11] in which SARS-CoV-2 spike glycoprotein-binding proteins, e.g., ACE2-like protein, are expressed by commensal bacteria of URT.

However, it must be considered that the interaction between bacteria and SARS-CoV-2 does not necessarily occur with native viral surface proteins, but most likely with a surface reworked by proteases and toxins [6] produced by bacteria. A likely explanation for this novel interaction between bacteria and coronaviruses could be based on the highly dynamic process generated between bacterial products (toxins, metabolytes, oligopeptides, proteases) and viral proteins. In addition, we have observed lytic plaques using bacterial species of the genus Dorea (see Figure S2 of Supplementary Materials) and have made the method we have described accessible to many researchers for further investigation. With this method is possible to observe the increase in fluorescence microscope after 30 days of bacterial cultures of the proteins of viruses that could have bacteriophages behavior (see Figure S3 of the Supplementary Materials). Therefore, immuno-EM, combined with molecular analysis of viral growth [7], and complemented by a fundamental assay of bacterial culture medium in the presence of nitrogen (^15^N), are important steps to observe and understand the mechanisms of a behavior such as bacteriophages.

It must be also remembered that bacteria are found on oral, nasal, pharyngeal, alveolar, and intestinal mucous membranes and are often more numerous than epithelial cells. Even if a virus does not replicate in them, this does not imply that it will not cause discomfort through the bacterial flora of the human being. Understanding that a virus also binds to, interacts with, and infects bacteria and that fecal-oral transmission is an additional source of spread, completely changes the epidemiological scenario of SARS-CoV-2. It changes how to intervene, how to give policy input, how to treat COVID-19 patients at the onset of symptoms, how to prevent, and perhaps another possible solution could be the vaccine as Sabin did: *“an attenuated virus for oral administration”*, *probably justified by the need of the bacteria of the microbiota to interact with the virus.*

Understanding whether bacteria can engineer certain viruses, can contribute to new mutations, as we [6,7], and others have reported [26], can have their own defense mechanisms, can react by producing toxic molecules to defend themselves [6], whether that defense ceases or remains in perpetuity, requires a number of essential controls to be performed. These controls on bacterial interactions should not be underestimated if the pathogen causes so much social and community damage.

The pandemic began in December 2019, and the scientific community still does not agree on when the virus began spreading among humans and what is the best solution. It is crucial to consider that once we have observed the replication of this virus in bacteria and understood that the route of transmission is also fecal-oral, it could be possible to control the virus spread and contribute with other specific pharmacological therapies to support current vaccines in order to improve the vaccination campaign.

## 5. Conclusions

The control of prokaryotic cells as a reservoir source for some viruses represents an element that should be the basis of any diagnostic pathway regardless of whether one is convinced that it can occur or not. Our results obtained by Immuno-EM, embedded immunogold, and fluorescence imaging highlight a close connection between the coronavirus SARS-CoV-2 and bacteria of the intestinal microflora. SARS-CoV-2 has two mechanisms of action; it infects both the eukaryotic cell, as reported in the current literature, but it also infects the human bacterial flora (two bacteria are already identified, data in preparation) and Sabin’s solution, the attenuated vaccine for oral administration, could be a solution that could complement the vaccination campaign to exit definitively from this pandemic. Our results confirm the role of bacterial co-factors in the multiplication of COVID-19 coronavirus during this new epidemic confirmed by the efficacy of some previously described antibiotics [7,8] on the early phase of viral infection. Whether it also plays a role in long-term disease remains to be determined. In this case, we could consider the virus as a bactériophage with a lytic phase and a lysogenic phase. Furthermore, the presence of the nitrogen isotope ^15^N in viral proteins after infection of bacteria with SARS-CoV-2 could represent a new approach to study the interaction of RNA viruses with human microflora.

## Data Availability

Not applicable.

## Supplementary Materials

The following supporting information can be downloaded at: https://www.mdpi.com/article/10.3390/vaccines10050708/s1, Additional evidence for observations of bacteriophage behavior of SARS-CoV-2.
