# Supplementary material for: Could SARS-CoV-2 Have Bacteriophage Behavior or Induce the Activity of Other Bacteriophages?
Source: Vaccines (Basel). 2022 Apr 29;10(5):708. doi: 10.3390/vaccines10050708 (PMC9143435; doi:10.3390/vaccines10050708)
Supplement: Supplementary file 1 [file vaccines-10-00708-s001.zip › vaccines-1697764-supplementary.pdf]

vaccines

MDPI

Supplementary materials to the work:
Could SARS-CoV-2 have bacteriophage behavior or induce the activity of other bacteriophages?

Carlo Brogna [1,*], Barbara Brogna [2], Domenico Rocco Bisaccia [1], Francesco Lauritano [1], Marino Giuliano [3], Luigi Montano [4], Simone Cristoni [5], Marina Prisco [6] and Marina Piscopo [6,*]

Title: Additional evidence for observations of bacteriophage behavior of SARS-CoV-2

FIGURE S1

Figure S1 shows the results of pre-embedding Immuno-EM, performed at another facility, as a control for the post-embedding Immuno-Gold assay. Nucleocapsid SARS-CoV-2(N) protein (Abcam, #ab273167) antibody immunogenicity assay was performed with the GFP-tagged N protein on Hela cells. More details are showed in paragraph 2.3 of materials and methods.

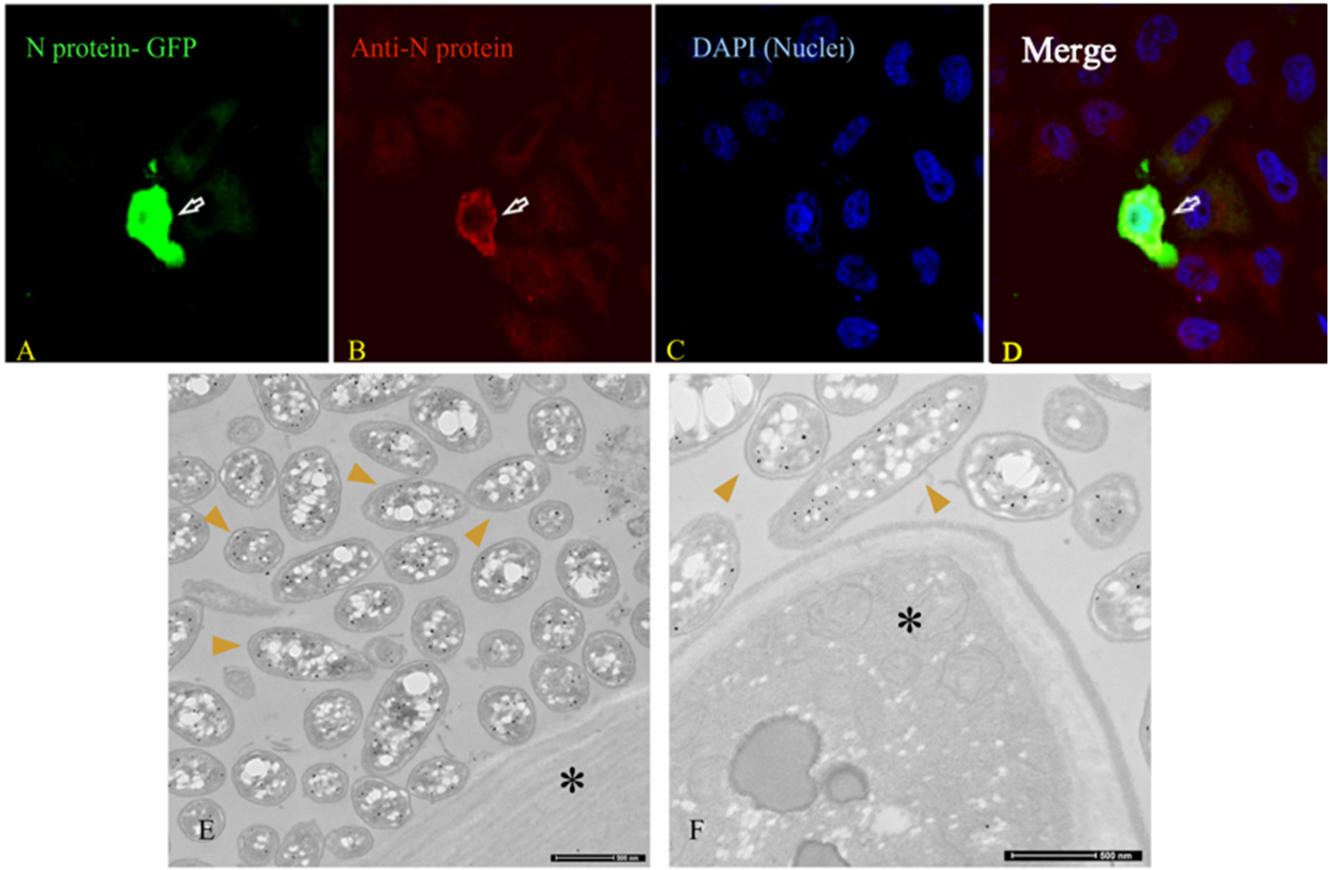

Figure S1: Panels A-D: Validation of antibody against the nucleocapsid protein of SARS-CoV-2. HeLa cells were transfected with GFP-tagged N protein of SARS-CoV-2, fixed, and labeled with antibody against GFP-tagged N protein of SARS-CoV-2. Arrow indicates cells expressing GFP-tagged protein N of SARS-CoV-2 (A); Arrow indicates the same cell in A labeled with antibody against protein N (B); ; DAPI on Hela cells (C); merge of A and B and C (D).**Panels E-F, immunogold pre-embedding**: show bacterial cultures with

antibody binding to protein N of SARS-CoV-2 around and within the bacteria. The asterisk (*) indicates the only yeast, eukaryotic, that we found in the cultures with no viral particles inside

TABLE S1

Table S1 shows the results obtained after 30 days of bacterial cultures, using methodology previously described in [7]. The table describes the operational taxonomic unit (OTU).In the column "B0" are counting the sequences-reads at the begging of the bacterial culture, while in the culumn "B1" are reported the reads after 30 days of bacterial culture in presence of SARS-CoV-2. The comparison between B0 and B1 shows that some bacterial genus tend to increase and others to decrease. These data suggest that the increase in viral load, as previously shown by Petrillo et al. [7], in bacterial cultures is associated with a decrease in some genera of bacteria after 30 days of bacterial culture in the presence of virus. This could indicate a lytic behavior of the virus towards these bacteria. Some Bacteria genus like *Dorea, Fusicatenibacter, Klebsiella, Streptococcusdecreased* while other bacteria genus like *Campylobacter, Prevotella, Staphylococcus, Bacteroides, and Citrobater* increased.

Table S1

Operational Taxonomic Unit (OTU)	B0 (panel A of figure 3)	B1 (panel B) after 30 days	0 norm	B1 norm	B0/B1	
Bacteria;*Actinobacteria;Actinobacteria;Bifidobacteriales;Bifidobacteriaceae;Bifidobacterium*	3902107	9784	0,28446632	0,00132104	215,334898	DECREASE
Bacteria;*Firmicutes;Clostridia;Clostridiales;Lachnospiraceae;Fusicatenibacter*	693939	2004	0,05058864	0,00027058	186,962828	DECREASE
Bacteria;*Proteobacteria;Gammaproteobacteria;Enterobacterales;Enterobacteriaceae;Klebsiella*	100159	770	0,00730166	0,00010397	70,2313383	DECREASE
Bacteria;*Firmicutes;Bacilli;Lactobacillales;Streptococcaceae;Streptococcus*	379044	5559	0,02763257	0,00075058	36,8149862	DECREASE
Bacteria;*Firmicutes;Clostridia;Clostridiales;Lachnospiraceae;Anaerostipes*	1516540	23327	0,11055682	0,00314963	35,1015803	DECREASE
Bacteria;*Firmicutes;Bacilli;Lactobacillales;Enterococcaceae;Enterococcus*	254447	6228	0,01854936	0,00084091	22,0587257	DECREASE
Bacteria;*Firmicutes;Clostridia;Clostridiales;Lachnospiraceae;Blautia*	3392831	141314	0,24733974	0,0190803	12,9630957	DECREASE
Bacteria;*Firmicutes;Clostridia;Clostridiales;Lachnospiraceae;Dorea*	355250	26718	0,02589797	0,00360748	7,17896491	DECREASE
Bacteria;*Firmicutes;Erysipelotrichia;Erysipelotrichales;Erysipelotrichaceae;Coprobacillus*	32916	3055	0,0023996	0,00041249	5,81738127	DECREASE
Bacteria;*Proteobacteria;Betaproteobacteria;Burkholderiales;unkn. Burkholderiales(o);unkn. Burkholderiales(o)*	1455	1638	0,00010607	0,00022116	0,47960178	INCREASE
Bacteria;*Firmicutes;Clostridia;Clostridiales;Clostridiaceae;unkn. Clostridiaceae(f)*	7504	8511	0,00054705	0,00114916	0,47604052	INCREASE

Bacteria;*Firmicutes;Clostridia;Clostridiales;Clostridiaceae;Hungatella*	1670	2319	0,00012174	0,00031311	0,38881894	
Bacteria;*Firmicutes;Tissierellia;Tissierellales;Peptoniphilaceae;Finegoldia*	1397	2376	0,00010184	0,00032081	0,31745463	INCREASE
Bacteria;*Firmicutes;Erysipelotrichia;Erysipelotrichales;Erysipelotrichaceae;Holdemanella*	2112	3849	0,00015397	0,00051969	0,29626319	INCREASE
Bacteria;*Firmicutes;Clostridia;Clostridiales;Ruminococcaceae;unkn. Ruminococcaceae(f)*	12378	30898	0,00090236	0,00417187	0,21629765	
Bacteria;*Proteobacteria;Epsilonproteobacteria;Campylobacterales;Campylobacteraceae;Campylobacter*	1820	4550	0,00013268	0,00061434	0,21596913	INCREASE
Bacteria;*Firmicutes;Clostridia;Clostridiales;Lachnospiraceae;unkn. Lachnospiraceae(f)*	361264	927144	0,0263364	0,12518353	0,21038229	
Bacteria;*Firmicutes;Clostridia;Clostridiales;Eubacteriaceae;Eubacterium*	189142	496358	0,01378858	0,06701855	0,2057428	INCREASE
Bacteria;*Firmicutes;Clostridia;Clostridiales;Clostridiaceae;Clostridium*	264066	924742	0,0192506	0,12485921	0,15417842	INCREASE
Bacteria;*Proteobacteria;unkn. Proteobacteria(p);unkn. Proteobacteria(p);unkn. Proteobacteria(p);unkn. Proteobacteria(p)*	2553	9210	0,00018612	0,00124354	0,1496659	INCREASE
Bacteria;*Firmicutes;Clostridia;Clostridiales;Lachnospiraceae;Roseburia*	110395	460112	0,00804787	0,06212459	0,12954407	
Bacteria;*Firmicutes;Clostridia;Clostridiales;Ruminococcaceae;Gemmiger*	38238	163891	0,00278758	0,02212866	0,12597134	INCREASE INC
Bacteria;*Firmicutes;unkn. Firmicutes(p);unkn. Firmicutes(p);unkn. Firmicutes(p);unkn. Firmicutes(p)*	90589	410401	0,006604	0,05541258	0,11917873	INCREASE
Bacteria;*Proteobacteria;Alphaproteobacteria;Rhizobiales;Methylobacteriaceae;Methylobacterium*	5259	31485	0,00038338	0,00425112	0,09018435	
Bacteria;*Bacteroidetes;Bacteroidia;Bacteroidales;Prevotellaceae;Prevotella*	9670	63278	0,00070495	0,00854383	0,08250978	INCREASE
Bacteria;*Bacteroidetes;Bacteroidia;Bacteroidales;Tannerellaceae;Parabacteroides*	4112	31881	0,00029977	0,00430459	0,06963905	INCREASE

Bacteria;*Firmicutes;Clostridia;Clostridiales;Oscillospiraceae;Oscillibacter*	3886	31012	0,00028329	0,00418726	0,06765575	INCREASE
Bacteria;*Firmicutes;Clostridia;Clostridiales;Ruminococcaceae;Faecalibacterium*	51098	422135	0,00372508	0,05699692	0,06535581	INCREASE
Bacteria;*Proteobacteria;Betaproteobacteria;Burkholderiales;Alcaligenaceae;Achromobacter*	12169	112782	0,00088713	0,01522789	0,05825682	INCREASE
Bacteria;*Firmicutes;Bacilli;Bacillales;Staphylococcaceae;Staphylococcus*	2220	28016	0,00016184	0,00378274	0,04278372	INCREASE
Bacteria;*Firmicutes;Clostridia;Clostridiales;Ruminococcaceae;Subdoligranulum*	1631	23057	0,0001189	0,00311317	0,03819292	INCREASE
Bacteria;*Bacteroidetes;Bacteroidia;Bacteroidales;Bacteroidaceae;Bacteroides*	25116	641063	0,00183097	0,0865567	0,02115346	INCREASE
						INCREASE
						INCREASE
						INCREASE
						INCREASE

vaccines MDPI

	INCREASE

FIGURE S2

Figure S2 shows lytic plaques on a plate where a bacterium of the genus *Dorea* (purchased from the Leibniz Institute DSMZ-German Collection of Microorganisms and Cell Cultures GmbH), was grown according to the seller's instructions and contaminated at different areas with different amounts of supernatant, derived from a 30-day bacterial culture and in which molecular testing confirmed the presence of SARS-CoV-2. More details on the growth methodology, contamination, aliquots, controls and sequencing of the virus in the plaque lysis zones were submitted to another journal by Petrillo et al. The image was obtained by kind permission of Dr. Brogna who is a major contributing author

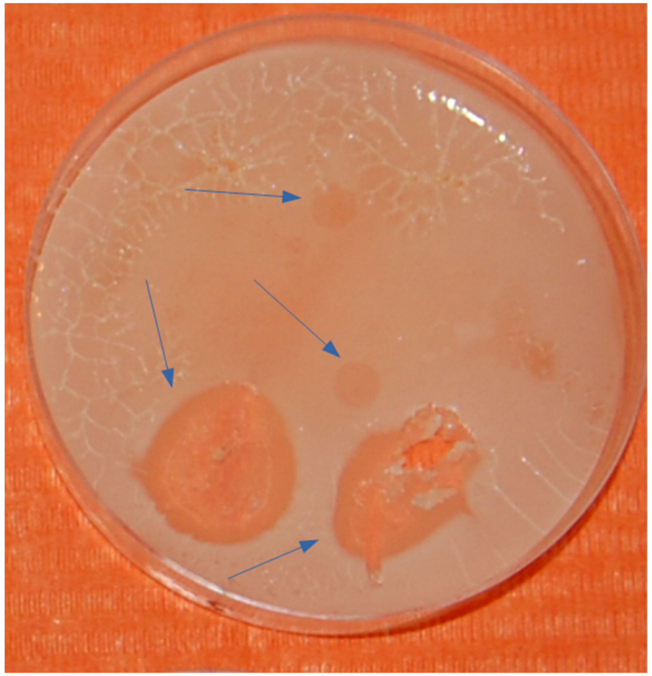

FIGURE S3

Figure S3 shows the immunofluorescence microscope images obtained with the method described in Petrillo et. Al. (7) to analyze the bacteriophagic behavior of viruses. In particular, we examined the microscopic fluorescence enhancement of a fixed aliquot (0.20µL) from day zero to day 30 of bacterial cultures in two samples A and B obtained as previously described by Petrillo et. Al. (7). An equal amount of culture broth was added after each withdrawal in order to not change the total bacterial culture volume. The immunofluorescence experiments -are described in the main document. In sample A were used as primary antibodies those versus spike SARS-CoV-2 protein (mouse monoclonal anti-SARS-CoV/SARS CoV-2 spike antibody; dilution 1:500, GeneTex, n. GTX632604-

vaccines MDPI

FDA approval) and as secondary antibodies the Goat anti-Mouse Alexa Fluor 488 (dilution 1:600, Invitrogen, #A28175- FDA approval) which generated the green light in sample A.

In sample B were used as primary antibodies those of nucleocapsid protein of SARS-CoV-2 and its secondary antibody (red signal in sample B) which are the same reported in material and methods, section immunofluorescence, in the main test. The method, which is for kind permission of Dr. Brogna, presented in another work and under patent, allows to evaluate with few laboratory or economic resources the activity of bacteriophages in general and of SARS-CoV-2 for our study. Fluorescence intensity enhancement was obtained with raph Pad Prism 9 software. Mean fluorescence images per sample were measured as integrated density on ImageJ software for analysis. Values are expressed as mean ±SEM. Statistical analysis was performed by one-way ANOVA comparing and data (P values <0.05 were considered statistically significant) observing that SARS-CoV-2 is also a bacteriophage.

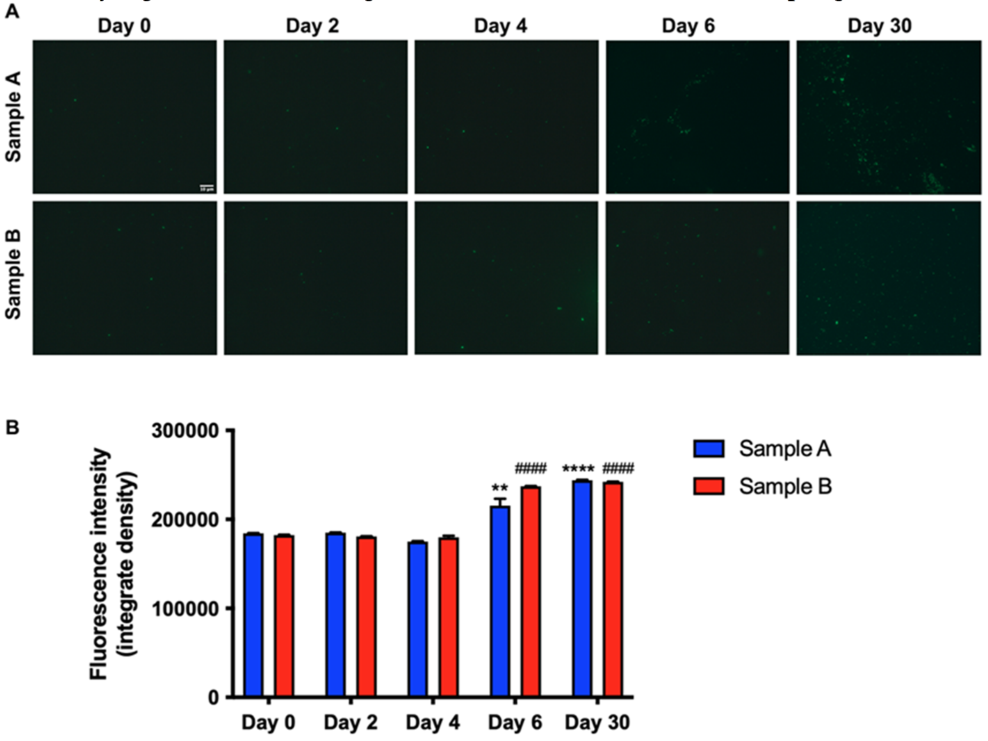

vaccines

MDPI

Figure S3: Immunofluorescence microscope images (Zeiss Axioplan 2, Axiocam 305 color, 100X magnification) on bacterial cultures (performed from day 0 to day 30) of positive cultures A and B, (for more details read Petrillo et al.(7). Fluorescence intensity increased in the two samples (sample A, green signal shows spike protein, and sample B, red signal shows nucleocapsid protein) from day 0 until day 30, observing the increase of viral proteins in the bacterial cultures.
